# Mechanisms of Heat Stress on Neuroendocrine and Organ Damage and Nutritional Measures of Prevention and Treatment in Poultry

**DOI:** 10.3390/biology13110926

**Published:** 2024-11-14

**Authors:** Yuyin Huang, Hongying Cai, Yunsheng Han, Peilong Yang

**Affiliations:** Key Laboratory of Feed Biotechnology of Ministry of Agriculture and Rural Affairs, Institute of Feed Research, Chinese Academy of Agricultural Sciences, Beijing 100081, China; yuyinhuang99@163.com (Y.H.); caihongying@caas.cn (H.C.); hanyunsheng@caas.cn (Y.H.)

**Keywords:** poultry, heat stress, neuroendocrine, organ damage, feed additives

## Abstract

Heat stress (HS) due to high temperatures has adverse effects on poultry, including decreased feed intake, lower feed efficiency, decreased body weight, and higher mortality. There are complex mechanisms behind heat stress in poultry involving the neuroendocrine system, organ damage, and other physiological systems. HS activates endocrine glands, such as the pituitary, adrenal, thyroid, and gonadal, by the action of the hypothalamus and sympathetic nerves, ultimately causing changes in hormone levels: HS leads to increased corticosterone levels, changes in triiodothyronine and thyroxine levels, decreased gonadotropin levels, reduced ovarian function, and the promotion of catecholamine release, which ultimately affects the normal productive performance of poultry. Meanwhile, heat stress also causes damage to the liver, lungs, intestines, and various immune organs, severely impairing organ function in poultry. Nutrient additives to feed are important measures of prevention and treatment, including natural plants and extracts, probiotics, amino acids, and other nutrients, which are effective in alleviating heat stress in poultry. Future studies need to explore the specific mechanisms through which heat stress impacts the neuroendocrine system in poultry and the interrelationships between the axes and organ damage so as to provide an effective theoretical basis for the development of preventive and treatment measures.

## Simple Summary

With global warming, heat stress has become an increasingly serious problem for poultry because of their abundant feathers, limited sweat glands, and fast metabolism. To alleviate the losses caused by poultry heat stress, it is important to investigate the mechanisms and control strategies for poultry heat stress. When heat stress occurs, the neuroendocrine system in poultry will be disrupted, while several organs in the body will be damaged, affecting the poultry’s production performance and health. Adding nutrients to feed is a mild and effective means of heat stress management and holds significant implications for the poultry farming industry.

## 1. Introduction

Heat stress (HS) has become a serious problem in poultry production due to global warming. Poultry are sensitive to heat stress because of their high metabolic rate, rapid growth rate, abundant plumage, limited sweat glands, and high farming densities [1,2]. The high-temperature environment is an important factor leading to stress and negative impacts on poultry [3,4]. HS could result in a decrease in feed intake, feed efficiency, body weights, and a significant increase in mortality in poultry [1,5], which reduces productivity and leads to substantial economic losses in poultry farming. Heat stress has diverse effects and mechanisms on poultry. The neuroendocrine system of poultry is disturbed under HS conditions, affecting growth and development, reproductive performance, and the immune response. In addition, HS leads to organ damage, including structural and functional abnormalities in the liver, intestines, lungs, and other organs. Various factors further affect the performance and health of poultry.

This review will highlight the mechanisms through which HS affects neuroendocrine secretion in poultry, summarize the organ damage caused by HS, and review nutritional prevention and treatment strategies in HS, with the aim of providing theoretical support for anti-HS research and breeding practices in poultry.

## 2. Heat Stress Response

The stress response refers to the behavioral and physiological reactions triggered by an animal facing internal and external stressors—a non-specific response aimed at maintaining homeostasis [6]. The stress response involves the collaboration of several body systems, including the endocrine system, the immune system, and the neurological system. Poultry are subjected to various forms of stress during the farming process, such as heat and cold stress, transport stress, slaughter stress, immune stress, and oxidative stress, all of which lead to significant economic losses in the farming industry [7].

High heat is one of the major stressors. When the thermal energy generated by the animal and the heat dissipation to the environment are imbalanced, heat stress (HS) develops. A variety of environmental factors (such as sunshine, heat exposure, climate, humidity, and exercise) as well as animal characteristics (such as species, metabolism, and thermoregulatory systems) combine to create this imbalance [8]. Thermal neutrality is the ideal temperature range for all homeothermic animals [9]. For broilers, the acclimatization temperature range is 18 to 25.9 °C [1], and HS occurs when temperatures exceed this range. HS can be broadly categorized into two types: acute heat stress (AHS) and chronic heat stress (CHS). AHS is caused by short bursts of extremely high temperatures, while CHS results from prolonged exposure to elevated heat [9]. Both forms of HS lead to varying degrees of mortality and declines in productive performance [10].

## 3. Heat Stress and Neuroendocrinology

**Figure 1:**
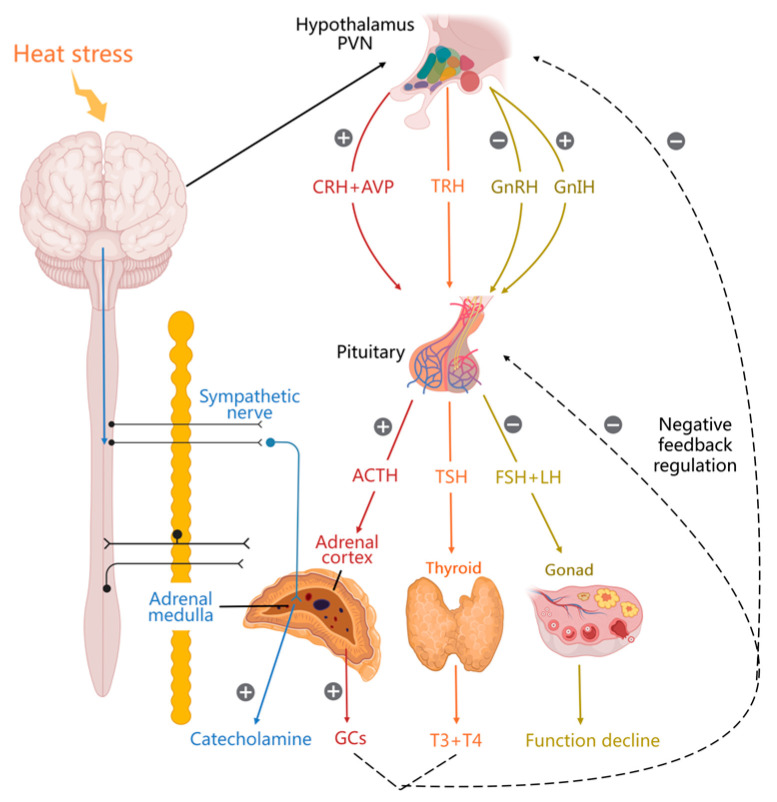
Effects of heat stress on the neuroendocrine system. + indicates upregulation, − indicates downregulation. (CRH: corticotropin-releasing hormone; AVP: arginine vasopressin; ACTH: adrenocorticotropic hormone; GC: glucocorticoid; GnRH: gonadotropin-releasing hormone; GnIH: gonadotropin-inhibiting hormone; FSH: follicle-stimulating hormone; LH: luteinizing hormone; TRH: thyrotropin-releasing hormone; TSH: thyroid-stimulating hormone; T3: triiodothyronine; T4: thyroxine).

Heat stress exerts various effects on animals, and the neuroendocrine axes—the hypothalamus–pituitary–adrenal, hypothalamus–pituitary–thyroid, hypothalamus–pituitary–gonadal, and sympathetic–adrenal medullary axes—have critical regulatory roles in mediating these effects (Figure 1). The main discoveries regarding the neuroendocrinology of heat-stressed poultry in recent years are outlined in Table A1 and reviewed in the subsequent sections.

### 3.1. Hypothalamic–Pituitary–Adrenal Axis (HPA)

**Figure 2:**
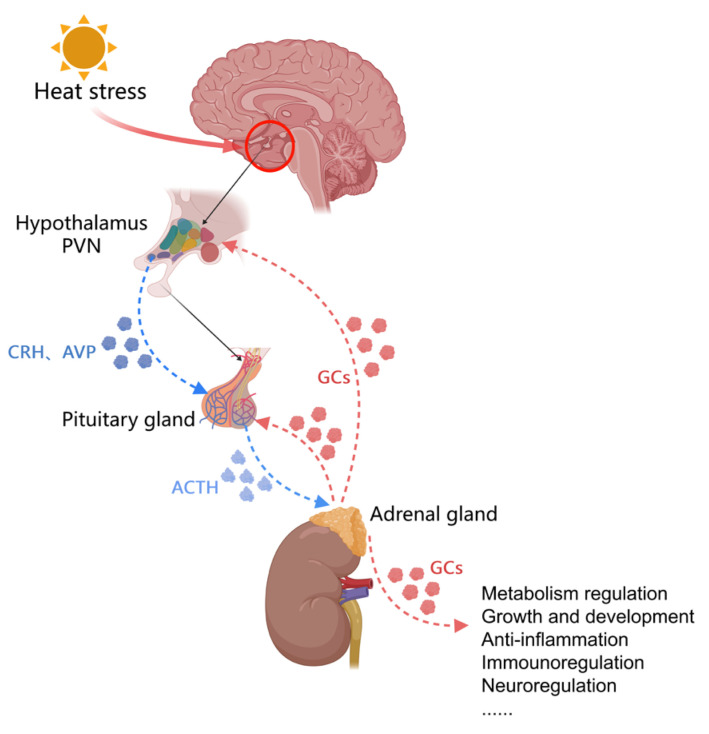
Mechanisms of the heat stress regulation of the hypothalamic–pituitary–adrenal axis. (PVN: the paraventricular nucleus of the hypothalamus; CRH: corticotropin-releasing hormone; AVP: arginine vasopressin; ACTH: adrenocorticotropic hormone; GC: glucocorticoid).

The hypothalamic–pituitary–adrenal axis (HPA) (Figure 2) is an important regulatory system in the stress response [11].

The hypothalamus functions as a central coordinator, responding to both exterior environmental cues (such as temperature) and interior biological indicators (such as hormones and enzymes) [12]. Thermoreceptors situated in the aorta and jugular arteries, which are responsible for monitoring body temperature, transmit signals to the hypothalamic control center [13]. The hypothalamic paraventricular nucleus (PVN) secretes arginine vasopressin (AVP) and corticotropin-releasing hormone (CRH), also known as corticotropin-releasing factor (CRF). Experiments have demonstrated that exposing broilers to high temperatures (32 °C for 7 days) significantly increased the concentration of plasma CRF [14]. Similarly, Li et al. [15] showed that heat stress caused an increase in the concentration of CRF in laying hens.

The pituitary gland, located beneath the hypothalamus, serves as the central gland of the endocrine system, synthesizing and secreting hormones [16]. CRH enters the pituitary portal vasculature and attaches to the CRH receptor (CRHR) on the pituitary gland (mainly CRHR1), activating the cAMP pathway, which stimulates the pituitary gland to secrete adrenocorticotropic hormone (ACTH) [11]. AVP is not only secreted into the pituitary portal system similarly to CRH but also is released from the axon terminals of the posterior pituitary gland, which synergistically enhances ACTH release in combination with CRH [11]. Beckford et al. [12] revealed that heat stress (35 ± 1 ℃ for 8 h) significantly reduced CRHR1 mRNA levels in the pituitary gland of broiler chickens [12].

The adrenal cortex binds to ACTH, subsequently synthesizing effector hormones, glucocorticoids (GC). Upon entering the bloodstream, ACTH functions in the adrenal cortex to induce the production and release of GC [17]. ACTH attaches to the melanocortin type 2 receptor (MC2-R) in the adrenocortical fasciculus. MC2-R activation triggers the cAMP pathway, which in turn induces steroidogenesis and the secretion of glucocorticoids, mineralocorticoids, and androgenic steroids [18,19]. The main GC in humans is cortisol, while the main glucocorticoid in poultry and rodents is corticosterone (CORT) [12,19].

HPA axis activity is highly variable with pulsatile glucocorticoid secretion and a circadian rhythm [20]. Moreover, GCs have a negative feedback regulation of ACTH and CRH secretion to maintain the equilibrium of the HPA axis [17]. GCs act on both the mineralocorticoid receptor (MR) and the glucocorticoid receptor (GR), while endogenous GCs have a much stronger attraction to the MR compared to the GR [21]. The MR is responsible for the circadian rhythms of cortisol, and the GR is responsible for the negative feedback regulation of high levels of cortisol [21]. After GC binding, GR regulates the transcription of HPA components, either by attaching to glucocorticoid response elements (GRE) or interacting with transcription elements [17]. However, excessive or sustained stress may lead to the dysregulation of GR activity, which can result in HPA hyperactivity and lead to disease [22]. FK506 binding protein 5 (FKBP5) is a regulator of the GR. GR can reduce its activity in response to cortisol via the transcriptional induction of FKBP5, thus isolating GR inside the cytoplasm and keeping it out of the nucleus [23,24].

HS increases HPA activity and induces the secretion of corticosterone. It has been shown that HS causes a substantial elevation in ACTH and CORT levels in broilers [25,26,27]. In addition to serum corticosterone concentrations, HS also increases corticosterone concentrations in broiler feathers [28,29]. Conversely, a study has also found that heat stress does not lead to significant changes in serum corticosterone levels in broilers [30], possibly due to circadian rhythms, negative feedback, stress from non-heat factors, and so on [31]. Corticosterone will respond and recover more rapidly to heat stress in Japanese quail. It was shown that serum CORT levels in Japanese quail were significantly elevated by 30 min of heat stress (34 °C) treatment, whereas after two hours, there was no significant difference anymore [32].

Glucocorticoids have diverse effects and play important roles in glucose metabolism, inflammation, immunity, neurology, among other processes [33]. Li et al. [34] demonstrated that exogenous glucocorticoids could promote gluconeogenesis, increase blood glucose concentration, and inhibit intestinal absorption of blood glucose in broilers. Heat stress resulted in elevated plasma CORT levels in broiler chickens, which in turn triggers the degradation of muscle proteins and fatty tissue, promotes gluconeogenesis, and enhances stress tolerance [26]. Glucocorticoids additionally induce muscle atrophy and promote the breakdown of muscle glycogen into glucose [35,36]. Moreover, glucocorticoids can reduce the action of osteoblasts and increase the action of osteoclasts, leading to osteoporosis and pathological fractures [37].

Glucocorticoids can be transferred from the poultry mother to the fetus or egg, thus affecting the offspring phenotype, a phenomenon known as the maternal effect [38]. Oluwagbenga et al. [39] demonstrated that the injection of exogenous glucocorticoids into Peking ducks increased cortisol concentrations in egg whites after the cortisol implant. Moreover, Oluwagbenga et al. [40] showed that heat-stressed breeders produced offspring with enhanced HPA and fear responses.

### 3.2. Hypothalamic–Pituitary–Thyroid Axis (HPT)

**Figure 3:**
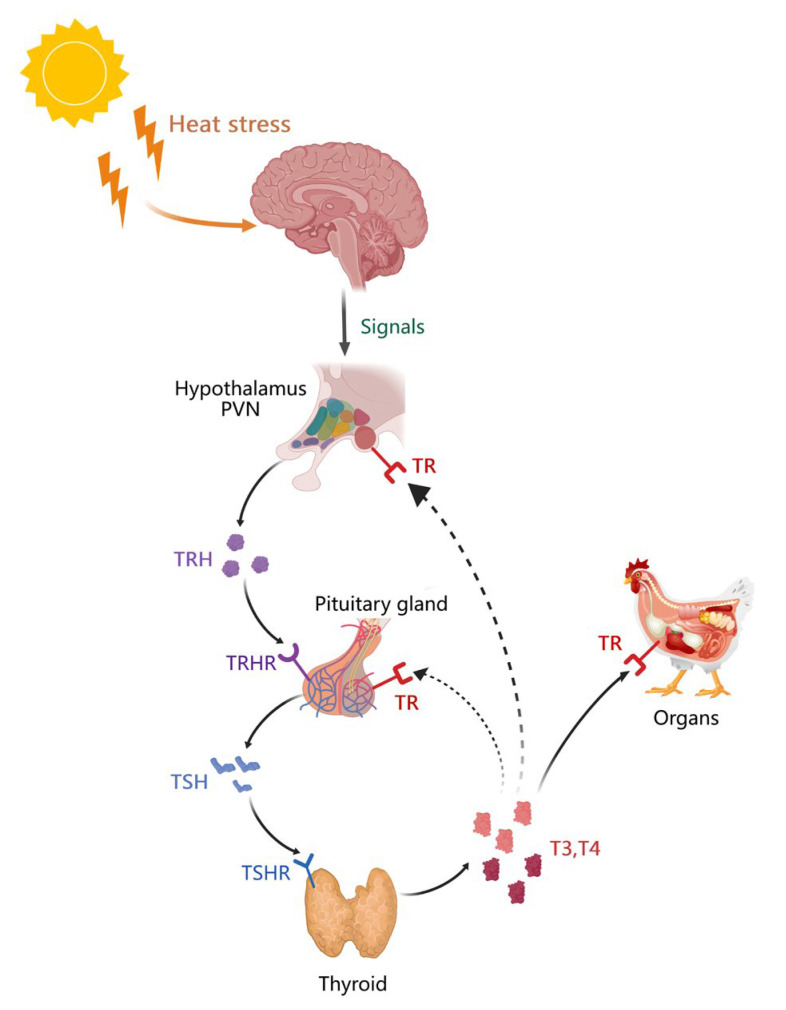
Mechanisms of the heat stress regulation of the hypothalamic–pituitary–thyroid axis. (PVN: paraventricular nucleus of the hypothalamus; TRH: thyrotropin-releasing hormone; TRHR: thyrotropin-releasing hormone receptor; TSH: thyroid-stimulating hormone; TSHR: thyroid-stimulating hormone receptor; T3: triiodothyronine; T4: thyroxine; TR: thyroid hormone receptor).

The hypothalamic–pituitary–thyroid axis (HPT) (Figure 3) is another critical stress-response system for animals [41]. The PVN of the hypothalamus receives afferent signals from various brain regions, primarily originating from internal environmental fluctuations or external stressors, and regulates the synthesis and release of thyrotropin-releasing hormone (TRH) [42]. TRH binds to the type 1 receptor (TRHR1) in the anterior pituitary gland, inducing the secretion of thyroid-stimulating hormone (TSH) [43]. TSH travels through the bloodstream and attaches to TSH receptors (TSHR) on follicular cells in the thyroid gland, thereby promoting the synthesis and secretion of thyroid hormone (TH) [11,43].

Thyroxine (T4) and triiodothyronine (T3) are the two main thyroid hormones, with T4 being more abundant in the blood but less biologically active than T3 [11]. The enzyme deiodinase (DIOs) can convert T4 to T3 in the thyroid, liver, hypothalamus, pituitary gland, and other tissues [11,43]. T3 has a higher affinity for the thyroid hormone receptor than T4 and serves as a major regulator of metabolism and thermogenesis in vertebrates [41]. The thyroid hormone receptor (TR) consists of two main subtypes, TRα and TRβ [43]. T3 and T4 inhibit the expression of the pituitary TRHR gene and TSH β gene as well as TSH release via negative feedback control [44], thereby maintaining internal environmental homeostasis. T3 and T4 are vital for protein synthesis and metabolism in almost every organ, including the brain, playing critical roles in metabolism, stress regulation, thermoregulation, and nutrient metabolism [43,45].

Several variables determine the impact of heat stress on thyroid hormone concentrations. Heat stress usually results in a decrease in plasma thyroid hormone concentrations [46], reducing metabolic heat output and lowering maintenance energy needs to avoid additional heat loads [9,47], thereby facilitating better acclimatization of poultry to high temperatures. This mechanism demonstrates biological adaptation. It was found that chicks with high thyroxine levels had reduced resistance to heat stress and increased mortality [48]. However, studies are not in complete agreement on the effects of heat stress on T3 and T4 levels. Vesco et al. [49] showed that heat stress (38 °C for 24 h) caused a decrease in T3 levels in quail, which is consistent with the findings by Alaqil et al. [50] and Zhuang et al. [51]. Hatipoglu et al. [52] found that 42 days of heat stress (34–36 °C for 5–7 h/day) resulted in decreased plasma T3 concentration and no significant change in plasma T4 levels in broilers. Somewhat differently, Beckford et al. [12] found that acute heat stress (35 ± 1 °C for 8 h) decreased plasma T3 levels while increasing plasma T4 levels in broilers, probably because heat exposure affected the conversion of T4 to T3. Sohail et al. [53] showed that 21 days of chronic heat stress decreased both serum T3 and T4 levels in broilers. Interestingly, the opposite findings have also been observed. Meng et al. [25] showed that chronic heat stress enhanced T3 and T4 levels in the plasma of white feather broilers. However, El-Kholy et al. [54] and Bueno et al. [55] found that heat stress did not lead to changes in T3 and T4 levels. These results suggest that the impact of heat stress on the poultry HPT axis is influenced by several parameters, notably the degree and duration of stress, the time of sampling, and the age of the bird [56].

The HPT axis does not function independently, as it interacts with the HPA axis [57]. Groef et al. [58] observed that CRH could induce TSH secretion by binding CRH type 2 receptors (CRHR2) in the broiler pituitary, suggesting that the HPA and HPT axes are interconnected. Beckford et al. [12] showed that exposing broilers to acute heat stress (35 ± 1 °C for 8 h) resulted in an upregulation of mRNA expression of CRHR2, TRβ, and TSHβ in the pituitary gland; this heat stress also led to elevated plasma corticosterone and T3 levels.

### 3.3. Hypothalamic–Pituitary–Gonadal Axis (HPGA)

The hypothalamic–pituitary–gonadal axis (HPGA) consists of the hypothalamus, pituitary gland, and gonads (ovaries or testes), which are closely involved in reproduction. The hypothalamus secretes gonadotropin-releasing hormone (GnRH), which binds to receptors on the pituitary gland and promotes the secretion of gonadotropin, including follicle-stimulating hormone (FSH) and luteinizing hormone (LH) [57]. LH and FSH exert their effects on the gonads and serve as major regulators of gonadogenesis, gametogenesis, and steroidogenesis [57]. The gonads secrete steroids, including estradiol, progesterone, and testosterone, which provide feedback to the brain and pituitary gland to regulate the production of GnRH and gonadotropins, respectively [59]. The activity of the HPGA closely affects poultry production performance. It has been shown that the differential expression of HPGA genes affects the egg-laying performance of ducks [60].

In addition to GnRH, the hypothalamus releases gonadotropin-inhibiting hormone (GnIH). In 2000, Tsutsui et al. [61] extracted a decapeptide from the hypothalamus of the Japanese quail, which can suppress the secretion of gonadotropin and was therefore named gonadotropin-inhibiting hormone (GnIH). Since then, GnIH has been found in other avian, rodent, and mammal species and plays a role in inhibiting gonadotropin synthesis and release [62]. A study in 2012 showed that heat stress (35 °C for 24 or 48 h) significantly upregulated pre-mRNA transcription of GnIH in the hypothalamus of chicks, which may be responsible for the decrease in feed intake [63].

Multiple studies have demonstrated that heat stress negatively impacts poultry reproduction and reduces reproductive performance. Oluwagbenga et al. [64] found that the number of follicles in the mature ovary, egg production, and egg quality of Peking ducks decreased after chronic heat stress. Pu et al. [32] found that Japanese quail exposed to heat stress experienced a reduction in ovary weight, oviduct weight, the number of stratified follicles, and body weight. A study has also shown that acute heat stress (35–37 °C for 5 days) reduces the number of large yellow and hierarchical follicles in Hy-Line Brown laying hens [15]. Heat stress not only affects female poultry but also impairs the reproductive capacity of males. Acute heat stress at 38 °C for 4 h would cause decreased sperm motility, viability, and concentrations [51].

Heat stress affects the HPGA through different pathways. During heat stress, GCs exert negative feedback regulation on the hypothalamus and pituitary gland, leading to a decrease in GnRH secretion, which in turn results in a decrease of FSH and LH release from the pituitary gland [65,66,67]. Li et al. [66] demonstrated that laying hens exposed to chronic heat stress (35 ± 2 °C for 16 weeks) had decreased plasma concentrations of LH, estradiol, and FSH. Changes in gonadotropin levels lead to a decline in ovarian function, impairing steroid production, which ultimately reduces reproductive efficiency [65]. Prolactin also contributes to ovarian decline and fewer mature follicles by decreasing GnRH levels in the hypothalamus and inhibiting pituitary gonadotropin secretion [68]. Heat stress can further disrupt blood flow, leading to impaired ovarian function. Wolfenson et al. [69] observed altered patterns of ovarian blood flow in heat-stressed hens, and the decline in ovarian function may be because of reduced blood flow to the ovaries. Furthermore, research has shown that heat stress triggers the process of programmed cell death in follicular cells by activating the FasL/Fas and TNF-α systems, resulting in a reduction in follicle number [15].

### 3.4. Sympathetic–Adrenal Medullary Axis (SAM)

The sympathetic–adrenal medullary axis (SAM) also has a significant impact on heat stress. Stress triggers the activation of the SAM and HPA systems to increase the release of glucocorticoids and adrenal hormones, thus enabling the animal to cope with the challenge better [70]. When broilers are subjected to acute heat stress, the SAM axis is rapidly mobilized to increase catecholamine secretion within a short period [71]. Catecholamines, including norepinephrine (NE), epinephrine (EP), and dopamine, function as both neurotransmitters and hormones, of which NE and EP are collectively known as adrenal hormones.

Sympathetic cholinergic preganglionic fibers innervate the adrenal medulla. During sympathetic excitation, the preganglionic fiber endings release acetylcholine, which acts on *N*-type receptors on medullary chromaffin cells and causes the release of catecholamines [46,72,73,74]. The interaction of catecholamines with adrenergic receptors existing on the membranes of all visceral organs and smooth muscle cells leads to the activation of signaling pathways, which cause rapid heartbeat, deepened breathing, elevated blood glucose and blood pressure, and dilated pupils in animals [46,72,73,74]. Blood is redistributed from the internal organs to the skin for thermoregulation, and it increases blood glucose and free fatty acid levels. According to Li et al.’s study [71], serum dopamine and epinephrine concentrations in broilers were elevated by acute heat stress (34 ± 1 ℃ for 24 h) in accordance with the findings of Calefi et al. [75]. Lyte et al. [76] showed that hens exposed to heat stress deposited norepinephrine into the eggs. Levels of catecholamines in poultry have been utilized as a significant marker for assessing stress [77].

## 4. Heat Stress and Organ Damage

**Figure 4:**
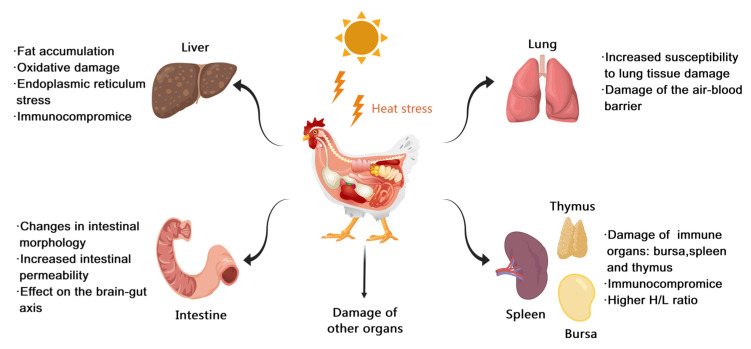
The organ damage in heat stress. (H/L ratio: the heterophil/lymphocyte ratio).

Heat stress can cause damage to many organs in the body along with neuroendocrine effects (Table A1), including the liver [78], intestines [79], lungs [80], and immune organs such as bursas of Falciparum [81] and spleens [82], thus affecting their normal function (Figure 4).

### 4.1. Liver

For poultry, the liver is the main site where fat is synthesized de novo [83]. Meanwhile, since birds lack a highly developed lymphatic system to absorb dietary lipids for circulation as mammals do [84], lipids ingested by poultry enter the liver in the form of particles [85]. The excessive synthesis of triglycerides by the liver leaves the poultry liver more susceptible to fat accumulation and liver damage [86]. Lu et al. [87] showed that chronic heat stress significantly increased the production of fat in the liver of broiler chickens and led to lipid deposition. High temperature interrupts the regular process of lipid metabolism and catabolism by downregulating enzymes participating in lipolysis, leading to increased fat accumulation and decreased protein in muscles [9,86]. Chronic heat stress also causes disruptions in the liver’s synthesis of bile acids, leading to abnormalities in lipid metabolism [88,89].

Heat stress causes a decrease in immunological function [90]. Studies showed that heat stress upregulated the expression of pro-inflammatory cytokines, downregulated the expression of anti-inflammatory cytokines, and increased inflammatory cell infiltration in the liver, thereby reducing liver weight and index, leading to liver damage in broilers [91,92,93,94].

Heat stress induces mitochondrial stress and oxidative stress in liver cells, while also inducing endoplasmic reticulum stress, leading to the disruption of the endoplasmic reticulum function and cellular homeostasis [95]. In their study, Jing et al. [96] provided evidence that prolonged exposure to high temperatures resulted in oxidative damage to the liver and led to the accumulation of lipids and infiltration of glycogen in broiler chickens. These effects were accompanied by dysfunction in the mitochondria, abnormal functioning of the tricarboxylic acid cycle within the mitochondria, and stress in the endoplasmic reticulum. Heat stress induces an increase in reactive oxygen species (ROS) and ultimately leads to oxidative damage in the liver [97]. Modern breeds of broilers and laying hens are particularly susceptible to heat stress because of their elevated mitochondrial metabolic rates [98]. The primary factors influencing the impact of heat stress on the liver of poultry are the compromised functioning of mitochondria and the subsequent effects on overall metabolism and energy balance [83]. Park et al. [99] showed that heat exposure increased oxidative stress in broiler livers, resulting in relatively slow growth rates.

Cyclic heat stress can improve the resistance of broilers to high temperatures. According to the study [100], in the broilers domesticated by cyclic heat stress, the gene expressions for ‘fatty acid degradation’ and ‘heat shock protein expression’ were upregulated, while the expression of genes for ‘cell cycle arrest’ and ‘amino acid metabolism’ was downregulated. They hypothesize that the domesticated group provided fat and energy to the tissues by breaking down fatty acids while also maintaining a stable internal environment via the use of heat shock proteins and antioxidant enzymes. Zinc (Zn) can mitigate the negative effects of heat stress on broiler livers by improving their ability to counteract oxidative stress and reducing the intensity of the heat shock response [101].

### 4.2. Enteric

The enteric system is very sensitive to heat stress. Prolonged heat stress negatively affects broilers’ performance, enteric morphology, and appetite, leading to a decrease in villus height (VH) and the ratio of VH to crypt depth (CD) [102], which can seriously affect intestinal health and lead to diseases such as enteritis and leaky gut [8,103]. Won et al. [104] found that heat-stressed chickens had lower intestinal villus height and higher intestinal permeability than controls. Ruff et al. [105] also found that heat stress increased intestinal permeability and induced gastrointestinal leakage. Heat stress induces changes in the structure of the small intestine, causing morphological damage and disorganization of the small intestinal epithelial cells in broilers, while different parts of the small intestinal villi are autolyzed in response to heat stress. Mehaisen et al. [106] showed heat stress decreased the depth, width, and surface of quail intestinal villi while increasing the depth of crypts; moreover, intestinal histology showed that heat stress resulted in the desquamation of the intestinal mucosal epithelium and severe destruction of the villi. The intestinal epithelial tight junction barrier serves a vital function in resisting pathogenic bacteria, endotoxins, and feed-associated antigens. During heat stress, decreased blood flow in intestinal epithelial cells and increased peripheral circulation lead to hypoxia, resulting in the breakdown of tight junctions, decreased intestinal integrity, and elevated intestinal permeability [107,108], ultimately increasing intestinal permeability to endotoxins, promoting the translocation of enteric pathogenic bacteria and increasing inflammatory cytokines in the serum [109].

The gut microbiota and the brain can establish bidirectional communication via the endocrine system and the neurological system, which is known as the brain–gut microbiota axis [110]. The gut microbiota could produce or stimulate the generation of neurotransmitters, such as serotonin, dopamine, and gamma-aminobutyric acid (GABA), which can affect the central nervous system [111]. The brain also influences gut function through neural signals and other mechanisms [112]. Nutritional levels have an impact on the development and functioning of the neurological system through the brain–gut axis, including the synthesis, release, and metabolism of thyroid hormones [56]. Calefi et al. [75] demonstrated that heat stress and necrotizing enteritis affected the levels of monoamines in various regions of the broilers’ central nervous system, particularly those associated with hypothalamic–pituitary–adrenal axis activity, providing evidence of a brain–gut axis. At the same time, nutritional levels affect the structure and function of the nervous system through the brain–gut microbiota axis, including the synthesis, release, and metabolism of thyroid hormones [56].

### 4.3. Lung

Poultry lacks sweat glands, and their bodies are covered with feathers, which affects thermoregulation. When temperatures are excessive, poultry dissipates heat primarily through respiration, and birds cool themselves by expanding their beaks to raise their breathing rate and promote respiratory evaporation [113]. The lungs are crucial respiratory organs that perform a vital function in maintaining the balance between oxygen and carbon dioxide and in controlling the blood’s pH. Shakeri et al. [114] showed that heat stress increased respiratory rate, decreased blood pCO_2_, and increased blood pH, indicating respiratory alkalosis in heat-stressed broilers, which ultimately led to reduced body weight. In addition, the surface of the lungs is rich in immune cells and chemicals like mucus, which act as a barrier against pathogens. Heat-stressed environments may lead to rapid respiration, which in turn increases the vulnerability to lung tissue injury [80]. Lu et al. [115] found that heat stress decreased the size of the alveolar sacs, clogged the alveolar septa, caused fluid buildup in the alveolar lumen, and facilitated the infiltration of inflammatory cells into the lungs. Heat stress also induced oxidative damage to the lungs and inhibited autophagy, which led to lung injury [115].

The primary pathological alteration in lung damage is the increased permeability of the air–blood barrier [116]. As the site of carbon dioxide and oxygen exchange, the air–blood barrier, also called the alveolar–capillary barrier, is an essential part of the lung functionality [117]. The disruption of the air–blood barrier can cause heightened lung permeability, hindered gas exchange, and the induction of dyspnea, which can result in diseases such as pneumonia [118]. Wu et al. [119] demonstrated that broiler lung tissue was harmed by heat stress due to the permeability and disruption of the air–blood barrier, which was exacerbated by the activation of the TLR/NF-κB signaling pathway, leading to heightened inflammation.

### 4.4. Immune Organs: Bursa of Fabricius, Spleen, and Thymus

Heat stress can suppress the immunity of poultry by damaging immune organs, including the bursa of Fabricius, spleen, and thymus. The bursa is situated dorsally to the cloaca in poultry, which is an important immunological organ for the production of humoral immunity, and serves a critical function in the proliferation and differentiation of B cells in poultry [120]. Stress can cause weight loss and reduced bursa function, which affects its immune function [121]. The thymus, located in the cervical region of poultry, participates in the maturation of T lymphocytes. Heat stress leads to oxidative damage to the immune organs and decreases the organ indices. Hirakawa et al. [122] showed that heat stress severely damaged the morphology of the thymic cortex and the bursa of the thymus, which affects the proliferation and functional advancement of T and B cells, leading to abnormalities in immunity in broilers. Research has demonstrated that heat stress has an impact on the immunological function of broilers by increasing the rate of bursal cell apoptosis and the cytokine levels in the bursa [81]. The spleen, another key immune organ in poultry, is involved in both humoral and cellular immunity, filtering out pathogens and damaged erythrocytes, and participating in erythrocyte production [65]. Mao et al. [82] found that heat stress could cause pathological damage to the spleen and thymus in broilers, reduce immunoglobulin content and antibody levels, and lead to oxidative damage in these organs.

Damage to immune organs by heat stress reduces immunocompetence in poultry. Research has demonstrated that HS inhibits humoral and cell-mediated immune responses in broilers, and heat stress significantly reduces the stimulation indices of T and B lymphocyte proliferation as well as antibody titers against sheep erythrocytes [123]. The heterophil/lymphocyte (H/L) ratio can serve as an indicator of the immune response in stressed poultry [124]. Heterophils are phagocytes that are in charge of the body’s innate defense against microbes, whereas lymphocytes have a unique function in adaptive immunity. Heat stress has been proven to increase the H/L and decrease immunity in broilers and laying hens [125].

### 4.5. Neuroendocrine System, Immunity, and Organ Damage

**Figure 5:**
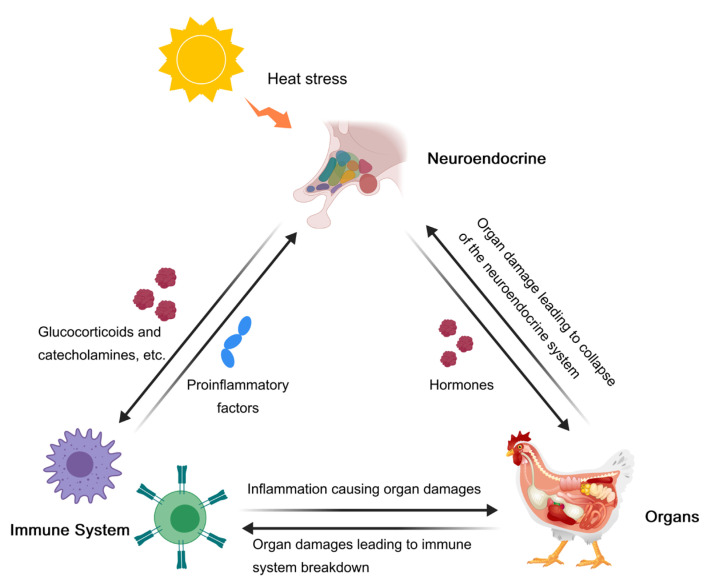
Interactions between the neuroendocrine system, immunity, and organ damages.

During heat stress, the neuroendocrine system, immunity, and organ damage interact with each other to form a complex physiological network. They interact and regulate each other, creating a series of dynamic changes from an initial adaptive response to possible pathological damage at a later stage (Figure 5).

When the temperature rises, temperature sensors in the skin and viscera send signals to the hypothalamus, and the neuroendocrine system is the first to kick in to try to suppress the inflammatory response. The activation of the HPA and SAM axes will promote the secretion of corticosterone and catecholamines, which will stimulate the production of macrophages [126], inhibit the expression of pro-inflammatory factors (including IL-2, IL-6, IFNγ, TNF, etc.) [127], and induce cellular and humoral immunity, resulting in immunosuppression and anti-inflammation [128]. In turn, pro-inflammatory cytokines stimulate neuroendocrine organs, including the hypothalamus, pituitary gland, and adrenal glands, thereby increasing hormone levels and further suppressing the inflammatory response [128]. However, persistent or severe heat stress disrupts the regulation of the immune system by the neuroendocrine system, and the immune response is over-activated, triggering systemic inflammation. The inflammatory response leads to lower relative weights of the thymus, bursa, spleen, and liver, which reduces immune function [8]. The relationship between inflammation and organ damage is often a vicious cycle of interaction and aggravation of each other. When the body is inflamed, a large number of pro-inflammatory factors are produced, leading to local tissue and organ damage, and the damaged tissue further activates the immune system, leading to the continued spread of inflammation [129]. For example, under heat stress, the intestinal barrier function is impaired, and intestinal bacteria as well as their metabolites (e.g., endotoxins) can enter the blood circulation, further activating the systemic inflammatory response and exacerbating multi-organ insufficiency [8]. If inflammation and organ damage continue to accumulate, the body may eventually enter a systemic inflammatory response syndrome (SIRS) [130]. At this point, the regulatory capacity of the neuroendocrine and immune systems is completely broken, and it is difficult for the organism to restore homeostasis, ultimately leading to irreversible multi-organ failure and even death.

In addition to the immune response, the neuroendocrine system also acts directly on the organs. Thyroid hormones have a significant impact on the processes of de novo lipogenesis, beta-oxidation (fatty acid oxidation), cholesterol metabolism, and carbohydrate metabolism [131]. Thyrotropin regulates hepatic bile acid homeostasis via the SREBP-2/HNF-4α/CYP7A1 axis [132]. Estrogen affects the liver as well. Estrogen receptor α (ERα) in the liver is a direct target of estrogen related to lipid and glucose homeostasis, growth, and development [133]. It has also been shown that endogenous GCs induce hepatic lipid accumulation and hypertriglyceridemia in pregnant mice by activating GR and its target gene, hepatic fatty acid translocase CD36, to accelerate fatty acid uptake in hepatocytes [134]. Additionally, catecholamines are involved in glycogenolysis and gluconeogenesis in the liver and play an active role in liver repair [135].

In conclusion, the neuroendocrine system, the immune system, and organ damage are closely related in heat stress, constituting a complex feedback loop. Short-term heat stress contributes to the regulation of metabolic and immune functions, which help animals adapt to high temperatures. However, prolonged heat stress can lead to immunosuppression and organ damage, affecting the function of multiple organ systems and disrupting the metabolic balance of the body. Understanding the interaction mechanisms is critical to developing effective measures. By optimizing environmental management, supplementing dietary additives (e.g., antioxidants, probiotics), and making appropriate pharmacological interventions, the negative effects of heat stress can be mitigated.

## 5. Nutritional Measures to Reduce Heat Stress in Poultry

**Figure 6:**
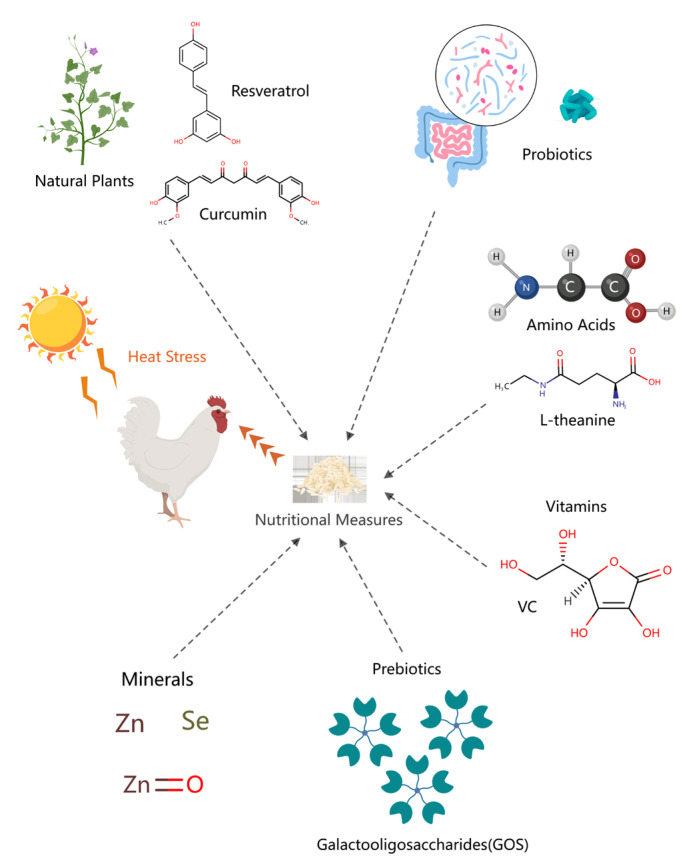
Nutritional measures to alleviate heat stress in poultry.

Nutritional regulation is an important prevention and treatment tool to mitigate heat stress in poultry, with the advantages of being effective and feasible, safe, and healthy. Among these, feed additives are effective anti-stress sources [136], including natural plants and extracts, probiotics, amino acids, and so on (Figure 6).

### 5.1. Natural Plants

Natural plants and extracts have the advantages of easy availability, high potency, and numerous biological effects [136]. Behboodi et al. [137] showed that a mixture of betaine, vitamin C, St. John’s wort, lavender, and melissa can combat heat stress by enhancing antioxidant capacity, reducing the elevation of corticosterone, and increasing the level of triiodothyronine. According to Wasti et al. [107], adding dried plums to the diets of broilers could ameliorate the decline in intestinal function induced by heat stress, which increased the expression of antioxidant genes, immune genes and tight junction protein genes in the gut, and improved intestinal microbial enrichment.

Polyphenols are common plant extracts that are characterized by a phenolic basic backbone with at least one aromatic ring carrying one or more hydroxyl groups [138]. Studies have shown that polyphenols are effective against heat stress. Polyphenols upregulate the production of heat shock proteins and antioxidant enzymes, thereby inhibiting ROS [139]. Two polyphenols commonly used in the defense against heat stress are summarized below.

Resveratrol (RES) is a naturally existing polyphenol chemical in a variety of plants [139] with anti-inflammatory and antioxidant properties [140]. Wang et al. [140] showed that RES improved intestine development and antioxidant function, leading to enhanced growth performance in heat-stressed broilers, which was mediated by the Nrf2 signaling pathway. Similarly, Ding et al. [92] found that RES improved the antioxidant activity of the liver and alleviated liver injury induced by high temperature by stimulating the Nrf2-Keap1 signaling pathway, thus enhancing the growth efficiency of heat-stressed broilers. Furthermore, RES can mitigate the neuroendocrine effects of heat stress. Meng et al. [25] showed that RES can alleviate the elevated serum levels of corticosterone, ACTH, and T3 induced by heat stress in broilers, with the features of improving intestine function, reducing the level of pro-inflammatory substances, and decreasing the rate of cell apoptosis.

Curcumin (feruloyl methane) is a yellow polyphenol compound derived from ginger, a kind of traditional Chinese medicine, which can mitigate the negative impact on performance resulting from heat stress, owing to its anti-inflammatory, antioxidant, and hormone-regulating properties [139,141,142]. In the study of Liu et al. [141], curcumin ameliorated the decline in laying performance and egg quality induced by heat stress in Hy-Line brown hens, improved antioxidant capacity, and increased the concentrations of FSH, LH, estradiol, IgG, IgA, and complement C. Zhang et al. [143] demonstrated that curcumin attenuated mitochondrial dysfunction, inhibited ROS burst, maintained thiol pools and mtDNA content, and enhanced the expression of mitochondrial antioxidant genes in heat-stressed broilers.

### 5.2. Probiotics

Probiotics are live microorganisms that manipulate and maintain beneficial microbial communities in the gut [144]. Probiotics can enhance the stability of the gut microbiota by suppressing harmful microbes, promoting the development of beneficial ones, and releasing immune regulators, thereby affecting the neuroendocrine system via the microbiota–intestinal–brain axis [145,146,147,148]. Dysbiosis of the intestinal flora can cause damage to the intestinal barrier, which results in endotoxemia and ultimately results in decreased production performance in heat-stressed poultry [149]. Feeding probiotics can improve the gut microbiota and the gut barrier, thus alleviating the adverse impacts of heat stress [149].

Mohammed et al. [150] demonstrated that the addition of synbiotics (consisting of *Bifidobacterium animalis*, *Enterococcus faecalis*, *Lactobacillus rohita*, *Lactococcus acidophilus*, and oligofructose) beneficially affected the cecum microbiota composition of heat-stressed broilers with a decrease in the harmful bacteria (*Escherichia coli*) and an increase in the number of viable beneficial bacteria (*Bifidobacterium* spp., *Lactobacillus* spp.), as well as an improvement in antioxidant capacity.

Prebiotics are also one of the important feeding additives that can selectively promote the proliferation and metabolism of probiotics in vivo, thereby altering the gut microbiota composition and improving host health [151]. Tavaniello et al. [152] showed that after injection of the prebiotic galactooligosaccharides (GOS) into fertilized eggs, hatched chicks were exposed to heat stress, and it was found that GOS improved production performance and increased collagen as well as monounsaturated fatty acid content in the pectoral muscle. Plant extracts can work together with probiotics to alleviate heat stress in poultry [153]. Elbaz et al. [153] found that feeding nutritional supplements (probiotics and clove essential oil) to broilers demonstrated a notable improvement in body weight increase, feed conversion, nutritive digestion, and digestive enzyme activity, with decreased abdominal fat, increased bursa weights, improved anti-inflammatory capacity, and increased height of ileal intestinal villi and depth of crypt fossa. This indicates that the supplement improves broiler growth performance, enhances immune antioxidant status, and alters the structure of the broiler’s ileum and its microbial community, thereby mitigating the negative impacts of heat stress [153].

### 5.3. Amino Acids

Amino acids can modulate the physiological functions of animals and play a significant part in alleviating heat stress in poultry [154]. Heat stress will lead to the production of large amounts of reactive oxygen species (ROS) in poultry, promoting oxidative stress, causing damage to the intestine and other organs, and ultimately affecting production performance [155]. The supplementation of feed with specific amino acids, such as sulfur amino acids, can reduce intestinal permeability and oxidative stress induced by heat stress to a certain extent [156].

Tryptophan is an important feed additive. Some products of tryptophan metabolism have antioxidant properties. For example, kynurenine and its derivatives can reduce damage from oxidative stress by scavenging free radicals or modulating redox reactions [157]. Thus, tryptophan metabolism may play a protective role in the fight against oxidative stress. Tryptophan can also regulate intestinal homeostasis by modulating the immune response through the aryl hydrocarbon receptor (AhR) signaling pathway [158]. A study has shown that supplementation of dietary tryptophan can alleviate oxidative stress and mitochondrial dysfunction, thereby alleviating heat stress in broilers [159].

A variety of non-conventional amino acids function significantly, which are generally metabolized derivatives of conventional amino acids. For example, *L*-theanine, a vital component of green tea, is a kind of non-protein water-soluble amino acid that can reduce the levels of corticosterone, dopamine, and norepinephrine, thereby serving as a natural component of stress resistance [160]. Saeed et al. [161] showed that supplementation of *L*-theanine in broiler diets could improve gut health by improving the synthesis of tight junction proteins (ZO-1, occludin, and claudin-3, among others) as well as by increasing beneficial microorganisms like *Lactobacillus* while decreasing harmful microorganisms such as *Clostridium difficile*. Zhang et al. [162] demonstrated that the dietary supplementation of *L*-theanine reduced indexes of the thymus, spleen, and Fasciola due to transportation stress, improved antioxidant capacity, and mitigated meat quality damage.

Additionally, there is an amino acid chelated with trace minerals, which provides both trace minerals and amino acids to improve nutrient utilization [163]. Baxter et al. [163] showed that the supplementation of amino acid chelated trace minerals could reduce corticosterone and decrease the expression of pro-inflammatory cytokines in the blood and jejunum, thus mitigating heat stress in broilers.

### 5.4. Nanomaterials

Nanotechnology is emerging as a new area of research in poultry nutrition. The intake of trace elements by poultry is inefficient, and it is easy to over-add, leading to a high dose of mineral elements being excreted in the feces [164]. The use of nanomaterials can improve the absorption and conversion rate, thus reducing environmental pollution [164]. Nano-trace elements or nano-nutrients (e.g., nano-selenium, nano-zinc, etc.) can increase the absorption rate of these nutrients in poultry and help them cope with heat stress due to their powerful antioxidant and immunomodulatory effects [165,166]. Huang et al. [167] showed by metabolomics that bioselenium nanoparticles could ameliorate heat stress-induced oxidative damage, metabolic disorders, and iron death in thigh muscles of broilers. In addition to nanometals, dietary addition of nano-propolis liposomes could increase heat tolerance by enhancing immunity, reducing inflammation and oxidative stress, and improving liver and intestinal structure in heat-stressed broilers [168].

### 5.5. Antioxidants

Compounds with strong antioxidant capacity (e.g., vitamins, minerals, etc.) have functions such as antioxidant activity, stress relief, immune regulation, anti-inflammation, intestinal protection, and growth promotion, making them effective additives for relieving heat stress in poultry [136]. The addition of VB6 to the diet could alleviate heat stress-induced reduction in the growth performance of broilers, reduce the level of inflammatory cytokines in the ileum, and simultaneously regulate the intestinal microbiota and metabolites to alleviate heat stress-induced damage to the intestinal barrier [169]. Pečjak et al. [170] demonstrated that the use of vitamin E, either alone or in conjunction with vitamins C and Se, has the potential to substantially enhance the α-tocopherol levels and decrease the malondialdehyde levels in the breast meat of broilers exposed to heat stress. Calik et al. [171] showed that the addition of vitamin E and Se in the diets significantly reduced the mRNA expression of heat shock proteins in the liver and jejunum, deregulated the mRNA levels of pro-inflammatory factors in the liver, and altered ileal microbiota composition along with its functions, thus alleviating the negative effects of heat stress in broilers. Feeding selenium-deficient diets induced multiple organ tissue damage with immune and redox dysregulation in broilers [172]. Hu et al. [173] demonstrated that under heat stress, the supplementation of dietary Zn increased the villus height and the villus width in the jejunum and ileum of xueshan chickens as well as improved serum antioxidant levels to alleviate the adverse effects of heat stress on broiler chickens. The dietary addition of the antioxidant compound ethoxyquinoline (EQ) could improve intestinal antioxidant capacity and immune response and thus reduce intestinal inflammation and improve productivity in heat-stressed broilers [174].

### 5.6. Nutritional Initiatives in Poultry Farming

In poultry farming, the use of feed additives has become an important measure to increase production efficiency, improve animal health, and optimize product quality. Additives significantly enhance feeding effects by supplementing nutrition, improving digestion, enhancing immunity, and in many other ways.

In antibiotic-free production systems, plant extracts and essential oils can act as natural growth promoters and immune enhancers. An appropriate addition of antioxidants as well as key amino acids to the feed can effectively mitigate the negative effects of heat stress on poultry growth and performance. During hot weather, electrolytes and vitamins, especially vitamin C and vitamin E, can be added to the drinking water to enhance the antioxidant capacity of the poultry and to maintain the electrolyte balance in the body. The effects of heat stress on intestinal health should not be ignored. Prolonged high temperature will lead to intestinal dysfunction. It is recommended to add probiotics and prebiotics in feed or drinking water, which can promote the growth of beneficial intestinal bacteria, maintain micro-ecological balance, and enhance the immunity of poultry.

Although additives can alleviate heat stress to a certain extent, environmental management is also important. The farming environment should be improved if possible, such as installing additional ventilation systems, using water spraying, or fogging cooling equipment combined with the use of additives to achieve better effects.

By following these specific recommendations, poultry producers can harness the full potential of feed additives to improve production efficiency, animal health, and product quality. The proper use of additives, tailored to the specific needs of the flock and environmental conditions, can significantly enhance the profitability and sustainability of poultry farming operations.

## 6. Discussion and Conclusions

With the increase in global temperatures and the rapid development of intensive farming, heat stress in poultry poses a serious challenge to the farming industry. Heat stress leads to neuroendocrine disorders and organ damage, affecting the growth, reproduction, and immune function of poultry. In recent years, researchers have extensively studied the mechanisms of the effects in heat-stressed poultry, mainly focusing on heat stress-induced oxidative stress, inflammatory responses, and effects on organs such as the intestines and liver. The effects and mechanisms of exogenously added nutritive or therapeutic substances have also been explored.

HS has an effect on poultry neuroendocrinology by the HPA, HPT, HPGA, and SAM axes. HS induces disorders in various neuroendocrine axes in poultry. Increasing CORT secretion through the activation of the HPA axis helps the body to cope with short-term heat stress, but prolonged stress may lead to metabolic disorders in poultry. Heat stress activates the HPT axis and causes changes in T3 and T4 levels, resulting in reduced body heat production and metabolism to adapt to the high temperature. During heat stress, there is a decrease in GnRH release and an increase in GnIH release, leading to a reduction in sex hormone levels and suppression of reproductive function. Heat stress promotes the body’s acute stress response by activating the SAM axis and increasing catecholamine secretion, but prolonged stress may lead to an increased burden on the cardiovascular system.

However, studies in recent years have mainly focused on oxidative stress, inflammatory response, and effects on organs such as the intestines and liver. Fewer studies have been conducted on neuroendocrine. Most of them were limited to the testing of hormone indexes or gene expression. There are few in-depth studies on the specific response mechanism of each axis under the influence of HS and relatively few studies discussing the interactions among the axes. Future studies could explore the effects of heat stress on animal metabolism, gene expression, antioxidant system, intestinal health, and endocrine immune regulation in depth through a variety of cutting-edge technologies, including metabolomics, proteomics, gene editing, and the microbiome. The brain–gut–microbe axis and the gut–liver axis are also important areas of research. Combined multi-omic research can comprehensively reveal the basic biological mechanisms of heat stress and will also provide an important theoretical basis for the development of new nutritional strategies and anti-stress measures.

Heat stress also affects poultry health by causing damage to several organs, including the liver, intestines, lungs, and immune organs, which is shown by metabolic disorders, impaired barrier function, respiratory dysfunction, and the suppression of the immune system. Prolonged or severe heat stress may lead to multi-organ dysfunction, exacerbate the body’s inflammatory response, and reduce resistance. However, most of the current poultry research explain these phenomena in terms of oxidative stress or immune stress but rarely combine the neuroendocrine axis with organ damage. There is still a gap in the studies with poultry, which researchers in the future can further investigate.

As climate change intensifies, future strategies to mitigate heat stress in animals should focus on a variety of novel nutritional interventions. Natural plant extracts have antioxidant and anti-inflammatory properties, which can be used to reduce oxidative stress and inflammatory responses associated with heat stress. Probiotics and prebiotics can significantly improve the gut health of animals by regulating the gut microbiota and maintaining gut barrier function. Amino acids (e.g., sulfur amino acids, tryptophan) and antioxidants such as vitamins (e.g., VC, VE) can strengthen the immune system and reduce cell damage. In addition, nano-nutrients are expected to be a breakthrough point for precision nutrition in the future due to their high bioavailability and targeting. These novel nutritional strategies combined with precision nutritional techniques will provide practical solutions to increase heat stress tolerance and improve production performance in poultry.

In conclusion, the mechanisms of heat stress in poultry on neuroendocrine and organ damage, as well as prevention and treatment, are an important and complex area. Future research could further explore the molecular mechanisms of heat stress, including how heat stress affects neurotransmitter release and signaling pathways and how it affects heat-stressed poultry through the neuroendocrine axis. Simultaneously, researchers can explore the mutual concerns between the axes and integrate the neuroendocrine axis with organ damage, leading to a deeper understanding of poultry health and providing more specific and accurate targets for the symptomatic relief of heat stress damage. There is a large gap in this area that deserves further exploration. Future research should continue to dive deeper into the uncharted territory of this field to provide better management strategies and solutions to ensure the sustainability of poultry production and animal welfare.

## Data Availability

Not applicable.

## Appendix A

**Table A1:** The main findings related to neuroendocrine in heat-stressed poultry.

	Heat Stress Treatment	Species	Main Findings About HPA	HPT	HPGA	SAM	Other Results About Organ Damage	REF
**CHS**	35 °C from 09:00 to 17:00 followed by 24 °C for the remaining time during 22–42 days of age	Male broiler chicks “Cobb500™”	·Significantly increased serum CORT	·Decreased serum T3			·Significantly increased serum H/L ·Increased serum AST and ALT	[50]
	34–36 °C for 5–7 h per day followed by 24 °C for 42 days	Mixed-sex Ross 308 broiler chicks	·Significantly increased serum CORT	·Decreased serum T3 ·No notable disparities in the levels of T4 and TSH.			·Significantly increased serum AST and ALT ·Significantly decreased total lactic acid bacteria (LABC) counts in cecal ·Significantly increased total *E. coli* count (EC) and total coliform bacteria (CBC) in cecal	[52]
	37 ± 2 °C for 8 h/d (09:00–17:00) followed by 24 ± 2 °C for 16 h/d during 28–42 days of age	White feather broilers	·Significantly increased CORT and ACTH concentrations in serum	·Significantly increased T3 and T4 concentrations in serum			·Decreased V/D in the jejunum ·Increased levels of apoptosis of the spleen ·Reduced splenic pro-inflammatory cytokine concentrations.	[25]
	32–36 °C during 14–42 days of age with different groups of period: ①heat stress from 11:00 to 12:00 ②from 11:00 to 13:00 ③from 11:00 to 14:00	Cobb Slow™ and Hubbard Flex™ strains male chicks		·No difference of T3 and T4 concentration ·Well-preserved thyroid morphology				[55]
	33 ± 2 °C during 2–6 weeks of age	Unsexed Japanese quail chicks		·No difference of T3 and T4 concentration and T3/T4 ratio				[54]
	34 °C for 4 h from 12:00 to 16:00 for 10 days	Female Japanese quail (*Coturnix japonica*)	·Significantly increased serum corticosterone after 30 min of heat challenge with no difference after 2 h		·Decreased ovary weight, oviduct weight, and hierarchical follicle number			[32]
	35 °C during 30–34 weeks of age	Hy-Line Brown laying hens	·Increased plasma CORT		·Decreased total egg number (EN), average egg weight (EW)		·Increased H/L ·Significantly declined the VH and CD of layer intestines ·Clear villi disruptions	[175]
	35 ± 2 °C during 22–38 weeks of age	Hy-Line Brown hens	·Significantly decreased CORT: Levels of CORT peaked at 26 wk of age, compared to that at 22 wk of age, and decreased until 38 wk of age.		·Significantly decreased the levels of LH, estradiol, and FSH			[66]
	34 °C for 28 days	Laying Fujian shelducks ducks	·Increased plasma CORT		·Less eggshell strength, eggshell thickness, and Haugh unit of the eggs ·Lighter yolk color ·Increased MDA content in yolk ·No differences in the egg shape index and albumen height ·Relatively reduced oviductal weights and shorter oviducts ·No differences in the number of large ovarian follicles ·Damaged tubular gland and the secretory mucosa cells of the magnum (oviduct) ·Interrupted connections between cells with the mucous lamina propria of the shell gland edematous in the ducks		·Evident necrosis of the intestinal epithelium	[176]
	35 °C for 10 h/d followed by 29.5 °C for 14 h/d from 85% lay for 3 weeks	Pekin ducks	·Significantly increased serum corticosterone and cortisol levels in female ducks, and no significant increase in male ducks		·Significantly decreased in egg production at weeks 1 and 3 ·Decreased fertile eggs upon candling at 10 days of incubation ·Decreased hatched chicks after the incubation period ·Increase dead embryo upon hatching ·Decreased number of maturing ovarian follicles in female ducks ·No significant differences in the relative weights of spleen or testes in male ducks			[64]
	35 ± 2 °C for 4 weeks	Jinding ducks			·Significantly decreased laying rate, total egg weight, average egg weight, yolk weight, eggshell weight, eggshell strength, yolk ratio, and eggshell thickness ·No significant difference in egg shape index, Haugh unit, or albumen height ·Heat stress affects the production performance and egg quality of Jinding ducks by regulating the secretion of endocrine-related hormones and the release of neurotransmitters as well as the expression of miRNAs and mRNAs in pituitary and ovarian tissues.			[177]
	34 ± 1 °C for 8 h per day for 18 days	Female Arbor Acres broilers	·Increased serum CRH			·Increased serum epinephrine	·Decreased bursal index	[178]
	34 ± 1 °C from 09:00 to 17:00 followed by 24 ± 1 °C for the remaining time during 22–42 days of age	Male broiler chicks (Cobb500™)	·Significantly increased serum corticosterone				·Significantly increased H/L ·Significantly increased activity ALT and AST as well as the levels of creatinine and urea, indicating negative impacts of heat stress on liver functions	[123]
	32 °C during 28–42 days of age	Arbor Acres male broilers	·Significantly increased CRF level after 7 d of heat exposure in plasma ·Significantly increased CORT level after 7 d and of heat exposure in plasma				·Significant increased panting after 7 d and 14 d of treatment ·Decreased thymus index after 7 d of heat exposure ·Lower organ indexes for the thymus and bursa of Fabricius after 14 d of heat exposure	[14]
	34 ± 1 °C from 09:00 to 18:00 followed by 26 ± 1 °C from 18:00 to 9:00 during 28–49 days of age	White recessive rock (WRR) chickens and the Lingshan (LS) chickens	·Significantly increased CORT concentrations in serum, with higher levels in the LS broilers				·Higher H/L in blood	[26]
	32 °C during 28–42 days of age	Male broiler chickens (Arbor Acres)	·Significantly increased CORT concentrations in serum after 7 days of heat exposure ·No significant difference in CORT concentrations in serum after 14 days of heat exposure				·Higher respiratory rates after 2 h, 3 D, and 7 D of heat exposure ·Increased relative weights of abdominal fat and liver	[86]
	34 ± 1 °C during 17–23 d of ages	Male broilers (Cobb 500)	·Increased serum CORT					[75]
	35 °C for 16 h per day during 28–42 d of ages	Male broilers (Cobb 500)	·Increased serum CORT				·Increased serum H/L	[179]
	34 ± 1 °C from 10:00 to 16:00 followed by 23 ± 1 °C for the remaining time during 22–42 days of age	Male broilers (Cobb 500)	·Increased serum CORT					[27]
	Two groups: 31 ± 1 °C and 36 ± 1 °C from 8:00 to 18:00 followed by 21 ± 1 °C for the remaining time during 35–42 days of age	Male broiler chickens	·Increased serum CORT				·Decreased relative weights of the spleen and thymus only in the HS36 °C group ·Decreased weight in the bursa of Fabricius in the HS31 °C and HS36 °C groups	[180]
	34 ± 1 °C from 9:00 to 17:00 followed by 22 ± 1 °C for the remaining time during 22–42 days of age	Male Arbor Acres broiler chicks	·Increased serum CORT				·Significantly decreased weights of breast muscle, liver, and spleen	[181]
	32 ± 1 °C from 10:00 to 16:00 for 9 weeks	Roman egg-laying hens	·Increased serum CORT				·Increased H/L ·Increased serum ALT activity ·Decreased liver weight	[182]
	31–32 °C for 8 h per day followed by 27–28 °C during 22–42 d of ages	Ross 308 male broiler chickens	·Increased feather CORT concentrations				·Increased blood H/L	[28]
	32 °C during 21–28 days and 30 °C during 29–35 days of age from 10:00 to 18:00, followed by 26 °C during 21–28 days and 24 °C during 29–35 days of age for the remaining time	Ross 308 male broiler chicks	·Increased CORT in feather					[29]
	35 °C from 8:00 to 16:00 during 24–38 days of age	Japanese quail (*Coturnix coturnix Japonica*)	·No difference in CORT concentration in male birds ·Higher urate sphere corticosterone metabolite concentration					[183]
	The temperature was increased from 24 °C at 8:30 during a 2-h period (about +0.1 °C/min) and maintained at 34 ± 1 °C until 16:30, then the temperature was gradually lowered returning to 24 °C approximately by 19:30 during 9 days. The following 3 days were used as a recovery period, reestablishing the thermoneutral temperatures (24 ± 2 °C).	Female Japanese quail	·No difference in CORT concentration				·Increased H/L	[184]
**CHS and AHS**	·Chronic heat stress treatment: 30 ± 2 °C for 10 days ·Acute heat stress treatment: 35 ± 2 °C from 09:00 to 13:00 followed by 20 ± 2 °C from 13:00 to 09:00 during 26–35 days of age	Hubbard classic broiler chicks	·Exposed to chronic heat stress: higher serum CORT levels on Day 3 ·Exposed to acute heat stress: the highest serum corticosterone levels on Days 3 and 10				·Both acute and chronic treatment: higher levels of serum endotoxins ·The highest prevalence of meat Salmonella infection in the chronic treatment in the liver followed by the acute heat stress treatment	[109]
**AHS**	Acute cyclical heat stress: 40 ± 1 °C from 10:00 to 14:00 for 7 days at 5 weeks of age	CARIBRO Vishal broilers	·Circulating corticosterone concentrations showed an inverse relationship concerning BW variation and HS duration	·Tri-iodothyronine concentrations showed an inverse relationship concerning BW variation and HS duration			·Reduced villi length and crypt depth	[185]
	35 ± 1 °C for 8 h at the age of Day 31	Ross 708 male broiler chicks	·Higher level of circulating CORT ·Decreased expression of corticotropin-releasing hormone receptor 1 (CRHR1) and increased expression of CRHR2 in the pituitary	·Significantly decreased plasma T3 by 62% and contrastingly increased circulating levels of T4 by 38% ·Increased expression of thyroid-stimulating hormone β (TSHβ) and thyroid hormone receptor β (TRβ) in the pituitary			·Higher mortality rate (20%) ·Higher blood pH ·Decreased pCO_2_ HCO_3_, K^+^, and total CO_2_ levels	[12]
	38 °C for 4 h at the age of 30 weeks	Broiler-type B-strain and 192 layer-type L2-strain TCCs roosters	·Increased plasma corticosterone levels	·Significantly decreased plasma T3 levels	·Decreased sperm motility, viability, and concentrations in both strains TCCs on Day 1 after the stress ·Recovered semen quality of the B-strain TCCs by Day 7 ·Lower semen quality of the L2-strain TCCs on Day 7 after the stress.			[51]
	35–37 °C for 5 days (S1–S5) followed by 24–26 °C for 15 days (R1–R15)	Hy-Line Brown laying hens	·Increased levels of corticotrophin-releasing hormone and corticosterone		·Decreased laying rates from day S3 to R6 ·Reduced numbers of large yellow and hierarchical follicles ·Triggered apoptosis while increasing the expression of FasL, Fas, TNF-α, and TNF-receptor 1 in small and large yellow follicles ·Decreasing the estradiol/progesterone ratio in follicular fluid in small and large yellow follicles.			[15]
	40 ± 0.5 °C for 2 h from 12:00 to 14:00	Wenchang chicks			·In female chicks, hypothalamic ERKs were upregulated in Weeks 1 and 2 while JNK and p38 were downregulated. ·Pituitary MAPKs were all downregulated in Week 3, but p38 was upregulated in Week 4. ·In the HS group, ovarian MAPKs were all upregulated during Week 5, whereas ERK was downregulated in Week 6. ·In contrast to the patterns of MAPK expression in female chicks, ERK in male chicks showed an opposite pattern in Weeks 1, 2, and 5, while p38 and JNK were downregulated in both female and male chicks under HS during Weeks 2 and 3. ·In the HS group, pituitary and testis MAPKs showed a pattern opposite to that observed in female chicks in Week 5; MAPKs were all downregulated.			[186]
	35 ± 1 °C for 3 h	Male broilers (Arbor Acres)	·Increased plasma CORT but with no statistical difference				·Significantly increased respiratory rate and rectal temperature	[187]
	34 ± 1 °C for 24 h	Female Arbor Acres broiler chicks	·Increased serum CORT, ACTH, and CRH			·Increased serum dopamine and adrenaline		[71]
	25 ± 0.9 °C for 6 days followed by 38 °C for 24 h	meat quails (*Coturnix coturnix coturnix*)		·Decreased T3 levels			·Decreased weight of the liver ·Lower lymphocyte percentage, greater eosinophil percentage, and increased H/L ratio	[49]
